# A retrospective comparison of helicopter transport versus ground transport in patients with severe sepsis and septic shock

**DOI:** 10.1186/s12245-016-0115-6

**Published:** 2016-06-07

**Authors:** Rahul Kashyap, Peter W. Anderson, Abhay Vakil, Christopher S. Russi, Rodrigo Cartin-Ceba

**Affiliations:** Department of Anesthesia and Critical Care Medicine, Mayo Clinic, 200 First Street, Rochester, MN USA; Multidisciplinary Epidemiology and Translational Research in Intensive Care (METRIC), Mayo Clinic, 200 First Street, SW, Rochester, 55905 MN USA; Department of Critical Care, Saint Alexius Medical Center, Bismarck, ND USA; Department of Pulmonary and Critical Care Medicine, Mayo Clinic, Rochester, MN USA; Department of Emergency Medicine, Mayo Clinic, Rochester, MN USA

## Abstract

**Background:**

Helicopter emergency medical services (HEMS) extend the reach of a tertiary care center significantly. However, its role in septic patients is unclear. Our study was performed to clarify the role of HEMS in severe sepsis and septic shock.

**Methods:**

This is a single-center retrospective cohort study. This study was performed at Mayo Clinic, Rochester, MN, in years 2007–2009. This study included a total of 181 consecutive adult patients admitted to the medical intensive care unit meeting criteria for severe sepsis or septic shock within 24 h of admission and transported from an acute care facility by a helicopter or ground ambulance. The primary predictive variable was the mode of transport. Multiple demographic, clinical, and treatment variables were collected and analyzed with univariate analysis followed by multivariate analysis.

**Results:**

The patients transported by HEMS had a significantly faster median transport time (1.3 versus 1.7 h, *p* < 0.01), faster time to meeting criteria for severe sepsis or septic shock (1.2 versus 2.9 h, *p* < 0.01), a higher SOFA score (9 versus 7, *p* < 0.01), higher incidence of acute respiratory distress syndrome (38 versus 18 %, *p* = 0.013), higher need for invasive mechanical ventilation (60 versus 41 % *p* = 0.014), higher ICU mortality (13.3 versus 4.1 %, *p* = 0.024), and an increased hospital mortality (17 versus 30 %, *p* = 0.04) when compared to those transported by ground. Distance traveled was not an independent predictor of hospital mortality on multivariate analysis.

**Conclusions:**

HEMS transport is associated with faster transport time, carries sicker patients, and is associated with higher hospital mortality compared with ground ambulance services for patients with severe sepsis or septic shock.

## Background

Helicopter emergency medical services (HEMS) significantly extend the reach of tertiary care facilities, leading to rapid transport of critically ill patients. A recent review of the literature on HEMS showed an overall benefit of 2.7 additional lives saved per 100 HEMS activations [1, 2]. For specific injuries/illness, the benefit of HEMS is clear. Trauma and ST elevation myocardial infarction (STEMI) comprise the vast majority of HEMS literature [2]. HEMS have been demonstrated to provide substantial benefit to patients suffering from trauma [3] and acute ST segment elevation myocardial infarction [4].

HEMS seem to provide the most benefit when there is a clear, time-sensitive therapeutic intervention(s) available at the receiving facility. With the advent of early goal-directed therapy, sepsis has become a “time critical” disease entity [5]. Unfortunately, not much is known about the efficacy of HEMS in sepsis, and intensivists at tertiary care facilities are forced to make the decision about the mode of transport without any evidence.

Until recently, HEMS transport of patients with stroke was considered to be beneficial. A recent single-center retrospective review of patients with acute stroke transported via HEMS versus ground transport after receiving recombinant tissue plasminogen activator failed to show benefit. The ability of the local emergency room physician to administer rTPA prior to transport is likely what negated the benefit [6]. It is unclear if the initial stabilization of the septic patient in the emergency room will have a similar effect.

Currently there is no data on the efficacy of HEMS transport in patients with severe sepsis or septic shock. Intensivists in tertiary care facilities are forced to make difficult and costly transport mode decisions without guidance from the literature. In this historical cohort, we aim to understand the effect on hospital mortality and other important secondary outcomes of helicopter transport in patients who develop severe sepsis or septic shock compared with ground transport.

## Methods

### Study subjects

After obtaining approval from our Institutional Review Board, we performed a retrospective chart review on 651 consecutive adults patients admitted to our medical intensive care unit during the years 2007–2009 with an established diagnosis of severe sepsis or septic shock. Severe sepsis and septic shock were established when patients had suspicion of infection and met any of the criteria: (1) a systolic blood pressure no higher than 90 mmHg (after a bolus of 20 mL/kg body weight fluid), (2) a blood lactate of 4 mmol/L or more, and (3) initiation of vasopressors [7–9].

Patients were included in the analysis if they were >18 years old, developed severe sepsis or septic shock within 24 h of hospital admission, were transferred from an acute care facility (hospital floor, intensive care unit, or emergency department), and were transported via ground ambulance or helicopter. Patients were excluded from the study if they did not have prior research authorization for their medical records to be reviewed; they had active bleeding, or they were started on comfort care patients within 12 h of admission.

The following data points were collected: age, gender, body mass index (BMI), distance traveled (defined as a one-way distance in miles), transport time (defined as time from outreach call being made to arrival at our hospital), time that patient met the criteria for severe sepsis or septic shock, Charlson score, Acute Physiology and Chronic Health Evaluation (APACHE) III score, sequential organ failure assessment (SOFA) score, fluid administered in the first 3 and 6 h after meeting the criteria for sepsis, initial antibiotic administration time, presence of acute respiratory distress syndrome (ARDS) (defined as *P*/*F* ratio less than 200 (1994 definition)) [10], need for invasive mechanical ventilation, time on ventilator, presence and stage of acute kidney injury (AKI) (from Acute Kidney Injury Network classification [11]), need for dialysis, intensive care unit (ICU) length of stay, hospital length of stay, ICU discharge status (alive or dead), and hospital discharge status (alive or dead). Adequate early goal-directed therapy (EGDT) was also assessed; this was defined as central venous oxygen saturation (ScVO2) ≥70 %, central venous pressure (CVP) ≥8 mmHg, mean arterial pressure (MAP) ≥65 mmHg, UO ≥0.5 mL/kg/h, and/or improvement in lactate within 6 h [3]. If resuscitation goals were not achieved within 6 h, then this was considered a failure to achieve EGDT.

### Statistical analysis

Continuous data are described as medians (interquartile range (IQR)) or means (standard deviation), as appropriate for non-parametric or parametric data, respectively. Categorical data are presented as counts with percentages. To test the difference in medians between groups, a Wilcoxon rank sum was used. Either the Fisher’s exact test or chi-squared test was used to note the differences in proportions where appropriate.

In order to evaluate the independent impact of transport time in hospital mortality, we created a multivariable logistic regression model a priori including transport time in hours, APACHE III 1 h as a marker of severity of illness, adequate EGDT and timing to adequate antibiotic therapy as markers of appropriate resuscitation. The final model was determined using both statistical and clinical criteria taking into consideration collinearity, interaction, and the number of patients who experienced the outcome of interest. The odds ratio (OR) and 95 % confidence intervals (CI) were calculated; *p* values of <0.05 were considered statistically significant. JMP statistical software (version 9.0, SAS institute, Cary, NC) was used for all analyses.

## Results

**Fig. 1:**
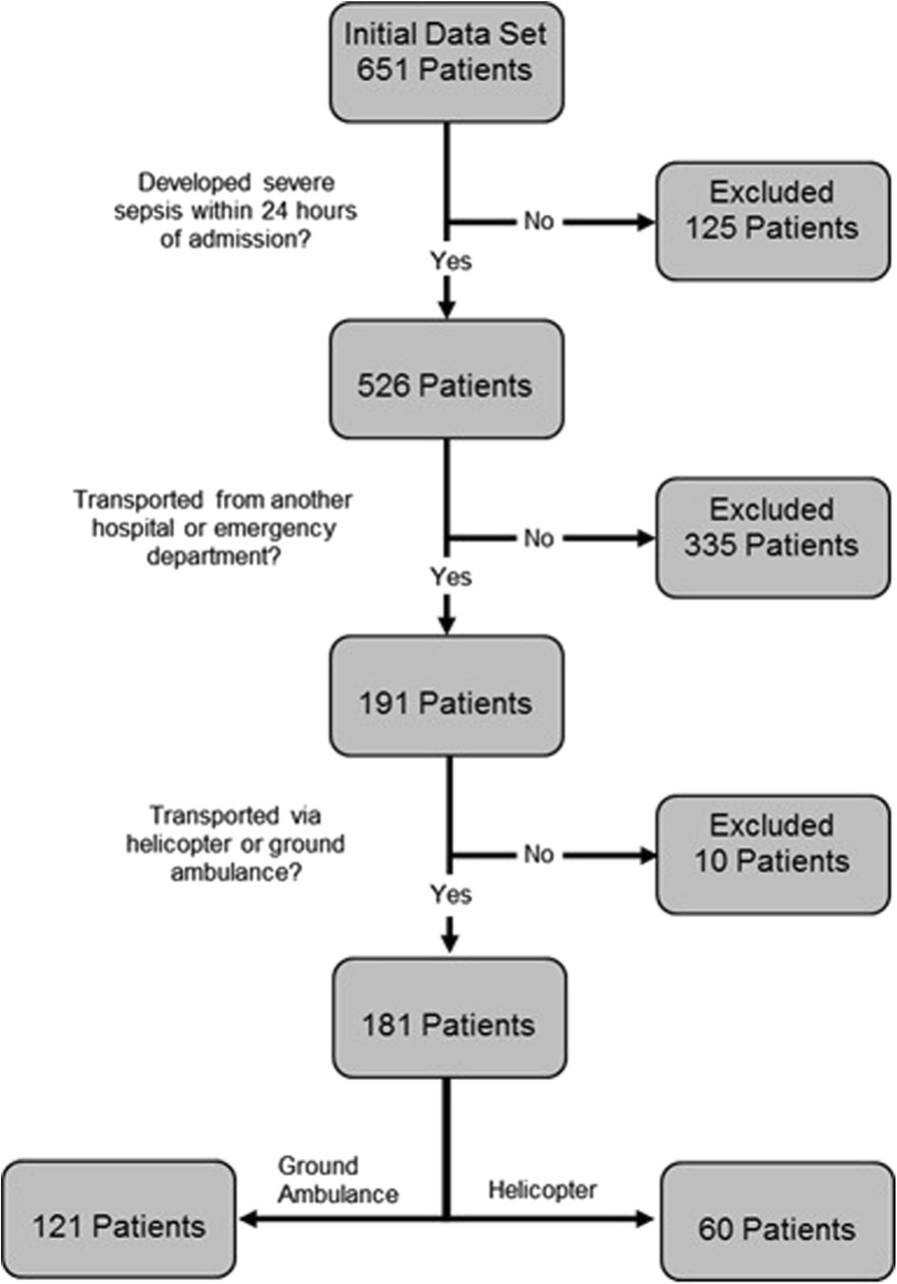
Consort diagram showing patient selection

Of the 651 patients who developed sepsis during our 3-year study period, a total of 181 (121 ground ambulance, 60 HEMS) meet our criteria for inclusion (Fig. 1). Both groups of patients were similar in initial characteristics (Table 1) except for decreased mean transport time (1.7 h for ground transport versus 1.3 h for HEMS), and patients transported via HEMS met the criteria for severe sepsis/septic shock after arrival to our institution sooner (2.9 h for ground transport versus 1.2 h for HEMS) and the median SOFA score at 1 day (median of 7 for ground transport versus 9 for helicopter transport). Both groups of patients received similar amounts of fluid resuscitation, received antibiotics in a similar time frame and were equally likely to meet EGDT goals.

**Table 1:** Initial characteristics of patients transported via ground ambulance and helicopter

	Ground ambulance (*N* = 121)	Helicopter (*N* = 60)	*p* value
Age (year), median (IQR)	73 (60–80)	64 (55–78)	0.06
Male, *n* (%)	62 (51)	35 (58)	0.36
BMI, median (IQR)	28.6 (25–35)	28.8 (24–34)	0.85
Initial creatinine (mg/dL), median (IQR)	1.0 (0.83–1.5)	0.9 (0.7–1.23)	0.12
Initial lactate (mmol/L), median (IQR)	1.7 (1.2–3)	2.2 (1.7–4.22)	0.13
Distance traveled (miles), median (IQR)	58 (44–67)	63 (44–86)	0.053
Transport time (h), median (IQR)	1.7 (1.4–2.3)	1.3 (1.1–1.8)	<0.01*
Time (h) from arrival to sepsis criteria met (h) median (IQR)	2.9 (1.5–6.4)	1.18 (0.6–3.5)	<0.01*
Charlson score, median (IQR)	5 (3–6)	5 (2–6)	0.18
APACHE 3 score, median (IQR)	58 (47–72)	63 (47–86)	0.18
SOFA score, median (IQR)	7 (5–10)	9 (6.2–12)	<0.01*
Fluid (L) first 3 h, median (IQR)	2.2 (1.0–4.1)	2.6 (1.2–4.6)	0.47
Fluid (L) first 6 h, median (IQR)	3.6 (1.7–5.9)	4.0 (2.2–6.2)	0.43
Time to antibiotics from sepsis criteria met (h), median (IQR)	0.25 (−1.35–1.18)	0.63 (−0.46–1.64)	0.073

*Statistically significant difference

From an outcome standpoint (Table 2), the patients transported via HEMS were more likely to develop ARDS (18 % for ground versus 38 % for HEMS), to require mechanical ventilation (41 % for ground versus 60 % for HEMS), to die in the ICU (4.1 % for ground versus 13.3 % for HEMS), and more likely to die prior to hospital discharge (17 % for ground versus 30 % for HEMS). Other outcome measures such as need for dialysis, AKI stage 2 or greater, and hospital and ICU lengths of stay showed no statistically significant differences.

**Table 2:** Outcome measures of patients transported via ground ambulance and helicopter

	Ground ambulance (*N* = 121)	Helicopter (*N* = 60)	*p* value
ARDS, *n* (%)	22 (18)	23 (38)	0.013*
Invasive mechanical ventilation, *n* (%)	50 (41)	36 (60)	0.014*
Ventilator days, median (IQR)	2.75 (1.3–5.8)	4.1 (0.9–7)	0.41
Acute kidney injury RIFLE 2 or more, *n* (%)	59 (49)	35 (58)	0.22
Need for dialysis, *n* (%)	26 (21)	18 (30)	0.21
Early goal-directed therapy goals met, *n* (%)	82 (68)	39 (65)	0.74
ICU length of stay, median (IQR)	2.8 (1.4–5.1)	4.1 (1.9–8.1)	0.051
Hospital length of stay, median (IQR)	8.9 (5.7–15.7)	10.9 (5.7–17.9)	0.31
ICU mortality, *n* (%)	5 (4.1)	8 (13.3)	0.024*
Hospital mortality, *n* (%)	20 (17)	18 (30)	0.04*

*Statistically significant difference

In the multivariate analysis (Table 3), transport time was found not to be an independent risk factor of hospital mortality after adjusting for covariates as specified in the methods section.

**Table 3:** Risk factors for hospital mortality in patients with severe sepsis and septic shock

	Odds Ratio	95% CI	*p* value
Transport time (hours)^❶^	0.77	0.48-1.13	0.22
APACHE III 1 hour^❷^	1.02	1.01-1.04	0.007
Adequate EGDT	0.38	0.17-0.82	0.01
Time to antibiotics (hours) ^❶^	1.07	0.94-1.25	0.32

❶For every hour

❷For every APACHE III point

*APACHE* Acute physiologic and chronic health evaluation, *EGDT* early goal directed therapy

A sensitivity analysis was performed for the patients who developed sepsis after 6 h of arrival to our hospital, and there was no difference in outcomes between ground transport and HEMS (data not shown).

Despite concern that weather may play a significant role in the decision to transport via HEMS or ground ambulance, we were only able to identify two instances in which the helicopter was unable to fly and the patients were transported via ground ambulance instead of helicopter.

## Discussion

Our data showed that, as compared to ground transport, HEMS transported patients presented a significantly faster median transport time, a faster time to meeting criteria for severe sepsis or septic shock after arrival, a higher SOFA score, higher incidence of ARDS, a higher need for invasive mechanical ventilation, higher ICU mortality, and increased hospital mortality. Multivariate analysis did not demonstrate that distance traveled was an independent predictor of hospital mortality; however, higher APACHE III scores and not achieving early goal-directed therapy goals were independent predictors of mortality. In addition, we found that there was no difference in survival in patients with sepsis transported via HEMS or ground transport. We showed that HEMS do improve transport time by 28 min. We also are able to reiterate that achieving early goal-directed therapy within 6 h is associated with improved outcomes.

Current guidelines regarding the mode of transportation in such patients are based on expert opinion and do not provide clear guidance. They leave the decision on transport mode to the clinicians involved in the transfer [12, 13]. The guidelines state that patients requiring a high level of care, but not suffering from a time critical illness, may be candidates for critical care ground transport, if available [14]. However, sepsis requires emergent intervention and early resuscitation with stabilization has shown to decrease mortality. In our study, the shorter transport time showed a trend towards decreased mortality but failed to achieve statistical significance. This may be due to a smaller sample size.

Several studies have evaluated the role of HEMS in the patients with trauma, acute STEMI, and stroke and in pregnant patients with obstetric emergencies, but none in patients with sepsis and septic shock. Taylor et al. [15] showed considerable variation in cost and effectiveness of HEMS in different clinical settings like trauma and acute STEMI. However, this variation can be attributed to the differences in the clinical health systems and health environments in which these studies were conducted. Nicholl et al. [16] compared the performance of an emergency helicopter and ground ambulances in a rural area in the UK and concluded that the transport times to hospital were 10 min faster by air transport than predicted for ground ambulance in trauma patients. This supports our finding of a faster transport time by HEMS. They also concluded that activation times, response times, and on-scene times were all longer on average for an emergency helicopter than for ground ambulance. However, it should be remembered that their study involved patients will all illnesses including trauma that needed higher degree of stabilization prior to transport. Our data lack activation times, response times, and on-scene times and thus cannot be compared. Studies conducted by Delgado et al. [17] and Falcone et al. [18] compared the effectiveness of HEMS with ground transport system for the transportation of trauma patients in the USA and failed to show benefit of HEMS for reducing mortality. Similar results have been shown by Olson et al. [6] in stroke patients requiring fibrinolytic therapy. Our study presents similar findings in patients with severe sepsis and septic shock.

On the other end, Kurola et al. [19] and Elliott et al. [20], who reviewed the effectiveness of HEMS in rural Finland and urban USA, showed the benefit of HEMS transport in patients with cardiovascular diseases and obstetric emergencies, respectively. However, this benefit can be attributed to the early availability of Advance Life Support care. These studies cannot be compared to our findings due to difference in the health care systems and different patient populations.

In an era of evidence-based medicine and given escalating health care costs, it is important to understand what benefit more expensive therapies offer patients. The cost of HEMS ranges from $6000–$25,000 per activation while ground ambulance transport cost is significantly less ($1200–$4000 per activation) [21]. Our data need to be validated in larger studies as this is important information for the clinician in order to make better informed decisions regarding mode of transportation for these critically ill individuals.

### Limitations

There are several limitations in our study. The sample size was small, and our data are from a single tertiary care center located in a mostly sub-urban area. Larger multicenter studies may overcome this limitation. Our results may not be generalizable to other centers where traffic, major civic events, or other urban concerns may limit the transport times of ground ambulances. Another notable limitation relates to how the diagnosis of sepsis was established. In our study, a patient was not declared septic until they met criteria for severe sepsis or septic shock at our institution. We do not have the data to ascertain if severe sepsis or septic shock diagnosis was made prior to transport and what therapies were given.

### Strengths

To the best of our knowledge, this is among the first studies to evaluate the role of HEMS in the patients with sepsis and septic shock. Our study builds on other important studies that have sought to clarify the added benefit of HEMS in various disease states.

## Conclusions

Our study shows that patients with severe sepsis or septic shock who are transported via HEMS have worse outcomes than patients transported via ground ambulance likely related to severity of disease rather than transport time. Patients transported via HEMS had a mean transportation time of 28 min faster than ground transportation; however, this time did not impact hospital mortality and other important secondary outcomes in our cohort. Larger multicenter studies are needed to fully understand the benefits of HEMS in patients with severe sepsis or septic shock.

## Abbreviations

AKI: acute kidney injury
APACHE: Acute Physiology and Chronic Health Evaluation
ARDS: acute respiratory distress syndrome
BMI: body mass index
CI: confidence intervals
CVP: central venous pressure
EGDT: early goal-directed therapy
HEMS: helicopter emergency medical services
ICU: intensive care unit
MAP: mean arterial pressure
OR: odds ratio
ScVO2: central venous oxygen saturation
SOFA: sequential organ failure assessment
STEMI: ST elevation myocardial infarction
